# Integration of DNA methylation and gene transcription across nineteen cell types reveals cell type-specific and genomic region-dependent regulatory patterns

**DOI:** 10.1038/s41598-017-03837-z

**Published:** 2017-06-15

**Authors:** Binhua Tang, Yufan Zhou, Chiou-Miin Wang, Tim H.-M. Huang, Victor X. Jin

**Affiliations:** 1 Epigenetics & Function Group, School of the Internet of Things, Hohai University, Jiangsu, 213022 China; 2 School of Public Health and Biostatistics, Shanghai Jiao Tong University, Shanghai, 200025 China; 3 Department of Molecular Medicine, University of Texas Health Science Center, San Antonio, TX 78229 USA

## Abstract

Despite numerous studies done on understanding the role of DNA methylation, limited work has focused on systems integration of cell type-specific interplay between DNA methylation and gene transcription. Through a genome-wide analysis of DNA methylation across 19 cell types with T-47D as reference, we identified 106,252 cell type-specific differentially-methylated CpGs categorized into 7,537 differentially (46.6% hyper- and 53.4% hypo-) methylated regions. We found 44% promoter regions and 75% CpG islands were T-47D cell type-specific methylated. Pyrosequencing experiments validated the cell type-specific methylation across three benchmark cell lines. Interestingly, these DMRs overlapped with 1,145 known tumor suppressor genes. We then developed a Bayesian Gaussian Regression model to measure the relationship among DNA methylation, genomic segment distribution, differential gene expression and tumor suppressor gene status. The model uncovered that 3′UTR methylation has much less impact on transcriptional activity than other regions. Integration of DNA methylation and 82 transcription factor binding information across the 19 cell types suggested diverse interplay patterns between the two regulators. Our integrative analysis reveals cell type-specific and genomic region-dependent regulatory patterns and provides a perspective for integrating hundreds of various omics-seq data together.

## Introduction

With the completion of second phase project of the Encyclopedia of DNA elements (ENCODE), thousands of regulatory elements within non-coding regions are now mapped and annotated within the human genome^1–4^. Thus, comprehensive understanding on their roles in mammalian development and human disease progression becomes increasingly important^5, 6^. DNA methylation, one of the key epigenetic modifications, plays crucial roles in mammalian cell differentiation, development, and proliferation^5, 7, 8^, and cancer initiation^9–11^, such as colorectal cancer^12^ and leukemia^13, 14^ and breast cancer^15–17^. Despite numerous studies done on understanding the role of DNA methylation, there is very limited work on systems integration of DNA methylation with gene expression and transcription factor (TF) binding across multiple cell types at a genome-wide manner.

In this study, we fully utilized the available ENCODE data resource, and conducted genome-wide integration of DNA methylation, TF binding, and RNA expression across 19 cell types. We first computationally identified cell type-specific differentially-methylated regions (DMR), then examined the genomic region specificity for these cell type-specific DMRs. We further developed a Bayesian regression model with Markov Chain Monte Carlo (MCMC) sampling technique to characterize the underlying statistical association among DNA methylation, genomic segment distribution, differential gene expression and tumor suppressor gene (TSG) status. We particularly examined the differential expression of TSGs in these regulatory regions since TSG is involved in many signaling pathways. The identification of TSGs and understanding their relations with DNA methylation are critical for further investigation of tumorigenesis^18–20^. We finally applied Kolmogorov-Smirnov (K-S) test in discriminative analysis of the background model and TF-specific methylation pattern^21^ to quantitatively examine the interplay of DNA methylation and TF binding.

## Results

### Examining cell type-specific patterns between array- and sequencing-based DNA methylation profiling

**Figure 1:**
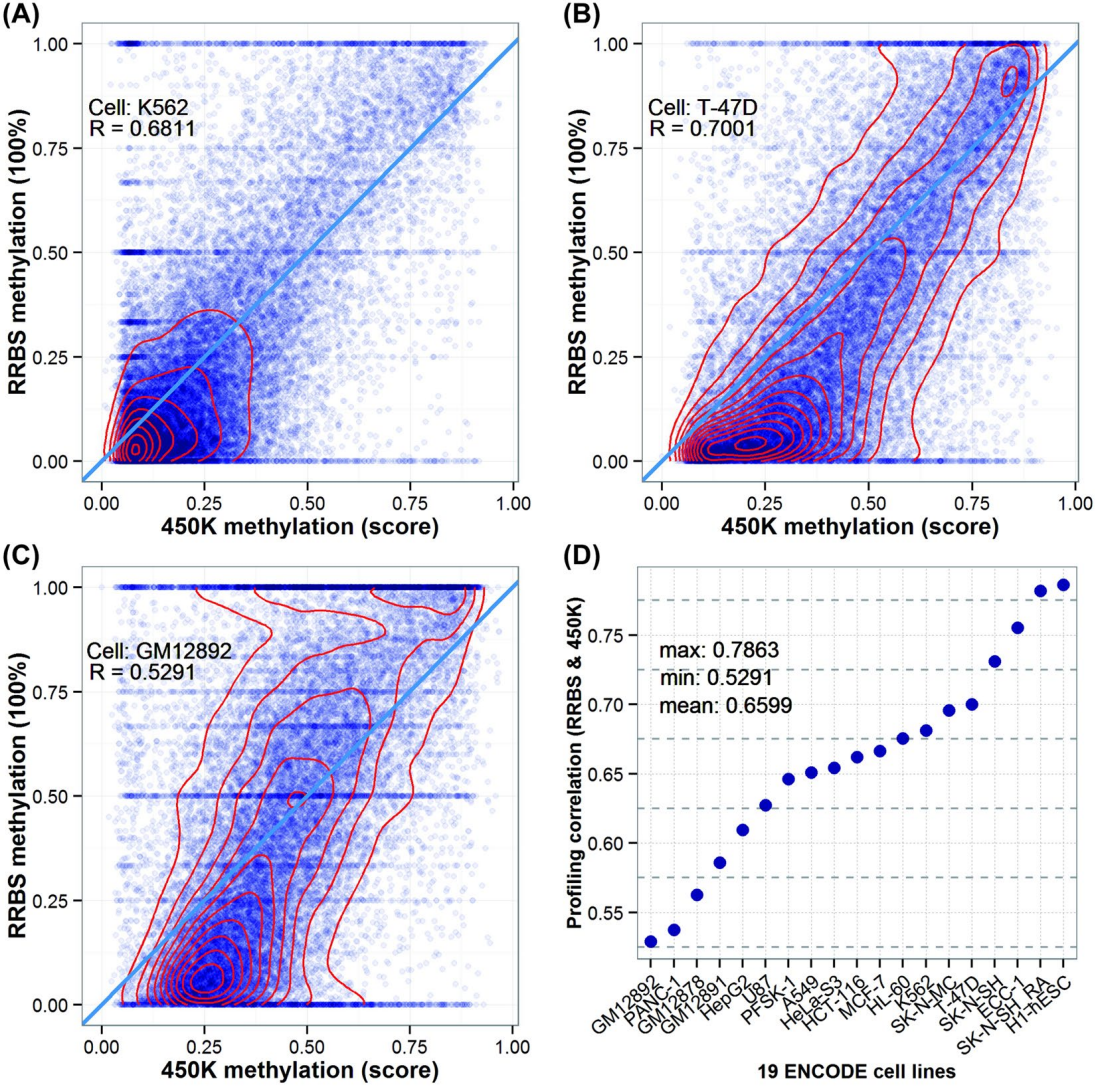
Genome-wide DNA methylation profiling comparison between two major platforms, Illumina Infinium Methylation Beadchip 450 K *vs*. RRBS. (**A**–**C**) Provide the illustrative examples for the three DNA methylation distribution patterns (unimodal, bimodal and trimodal) and corresponding *Pearson* correlation coefficient between two profiling platforms (top left corner); (**D**) presents the correlation statistics between two DNA methylation profiling platforms across the 19 ENCODE cell types.

To determine cell type-specific differential DNA methylation across 19 ENCODE cell types (Supplemental Material Table S1), we first examined the quantitative difference between two widely adopted platforms, array-based Illumina Infinium Methylation Beadchip 450 K^22, 23^, and sequencing-based reduced representation bisulfite sequencing (RRBS)^24, 25^. We performed the kernel density estimation on DNA methylation distribution to engender profiling contours and observed a clear unimodal, bimodal and trimodal distribution pattern for K562 (A), T-47D (B) and GM12892 (C) respectively (Fig. 1). Most other 16 cell types followed a bimodal pattern (Supplemental Material Figure S1(I)), except that GM12878 (Figure S1(I) C) and GM12891 (Figure S1(I) D) showed a trimodal distribution pattern and HepG2 (Figure S1(I) M) and PANC-1 (Figure S1(I) O) displayed a divergent distribution in a central section. Overall, the average correlation of two profiling platforms is 0.6599 (Fig. 1D), with a range from 0.7863 (H1-hESC) to 0.5291 (GM12892).

Interestingly, we found 450 K profiling tends to detect lowly-methylated CpG sites due to its probes scattered across the gene, while RRBS can identify highly-methylated CpG sites due to the restriction enzyme used particularly on enriched methylated regions. Our results are useful in determining which platform is suitable for experiment design, suggesting that RRBS is a choice for large-scale samples or population-based studies in despite of a relatively higher cost than 450 K.

**Figure 2:**
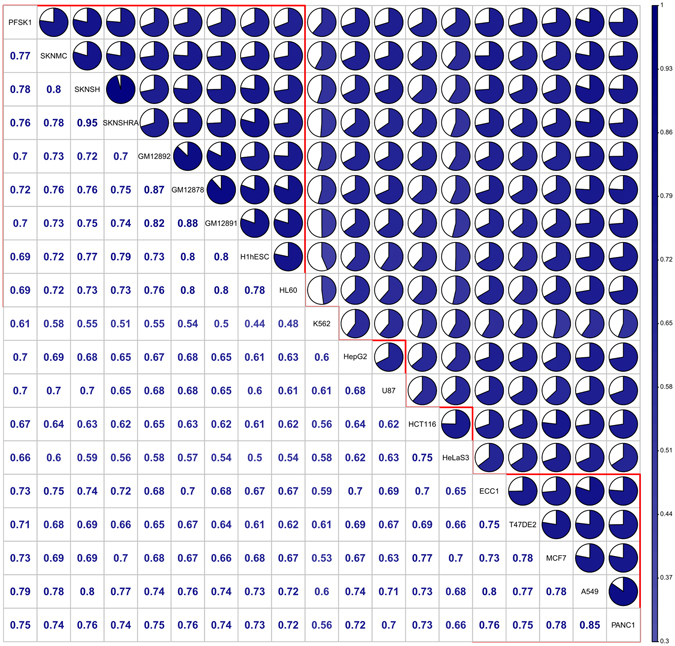
Genome-wide *Pearson* correlation chart for DNA methylation CpG base profiles across 19 cell types. Each diagonal entry gives cell name, upper off-diagonal entry denotes pie chart for the pairwise correlation level and lower off-diagonal entry denotes the corresponding pairwise correlation coefficient for genome-wide methylation of each cell line. The detailed corresponding methylation (Illumina Infinium 450 K) statistics for each cell line was in the Supplemental Material Section 2.

We further performed a pairwise correlation among any of two cell types using RRBS data and found an average pairwise correlation coefficient is 0.6908 (Fig. 2). We observed the following correlations: 1) Two breast cancer cell types, MCF-7 and T-47D, have a relatively higher correlation of 0.78; 2) A pancreatic cancer cell type, PANC-1, has higher correlation with some of solid cancer cell types, 0.78 with MCF-7, 0.75 with T-47D, 0.85 with a lung cancer cell type, A549, 0.80 with a endometrial cancer cell type, ECC-1, respectively; 3) Three brain tumor cell types, SK-N-SH, SK-N-MC and SK-N-SH_RA, have noticeably high pairwise correlations, from 0.78 to 0.95, but low correlation with two other brain tumor cell types, PFSK-1 and U87 (0.65 ≤ R ≤ 0.78); 4) Three blood cell types, GM12878, GM12891 and GM12892, have higher correlations with each other (R > 0.80) as well as with all other cell types except a leukemia cell type, K562 (R ~ 0.5) and a cervical cancer cell type, HeLa-S3 (0.54 ≤ R ≤ 0.58). This may be due to their trimodal methylation distribution patterns; 5) As expected, K562 has relatively lower correlations with all other cell types (0.44 ≤ R ≤ 0.61) partially due to its unimodal pattern (Fig. 1). Our results demonstrated that similar cell origins regardless of their morbidities (cancer or normal) have high correlations for their genome-wide methylation distribution patterns.

The histogram of CpG methylation distribution (RRBS and 450 K) for each of 19 cell types is shown in Supplemental Material Figure S2.

### Identifying cell type-specific differentially-methylated regions

Next, we sought to identify the differentially-methylated regions (DMRs) using RRBS data in 19 cell types, and further to interrogate their cell type specificity. Given the fact that 64.24% of CpG sites on average among 19 cell types were covered ≥ 10 sequencing reads, we only used those sites satisfying the sequencing depth with ≥ 10 reads for downstream analyses (see a detail of reads coverage for those cell lines in Supplemental Material Table S1).

As a demonstrated cell type, we selected T-47D as a reference since this ERα + breast cancer cell type is used in many studies in our lab and commonly used by other cancer researchers. We detected 688,445 differentially-methylated CpGs (DMCs), and further categorized them into 106,252 significantly DMCs, including 52,232 hyper- and 54,020 hypo-DMCs (threshold of absolute methylation difference ≥ 25%, adjusted q-value ≤ 0.01). Of them, 32% DMCs locate in promoter regions, 12% in exons, 25% in introns and 32% in intergenic regions; meanwhile, 56% of those DMCs distribute within CpG islands, and 12% at CpG islands shores. Interestingly, we found that promoter regions and CpG islands tend to be hypo-methylated in T-47D, containing 44% and 75% of total hypo-DMCs, respectively; and intergenic regions and CpG island shores only host 25% and 9% of total hypo-DMCs, respectively (Supplemental Material Figure S3). The results indicate that DMCs in promoter or CpG islands are more associated with cell type-specific transcriptional regulation.

**Figure 3:**
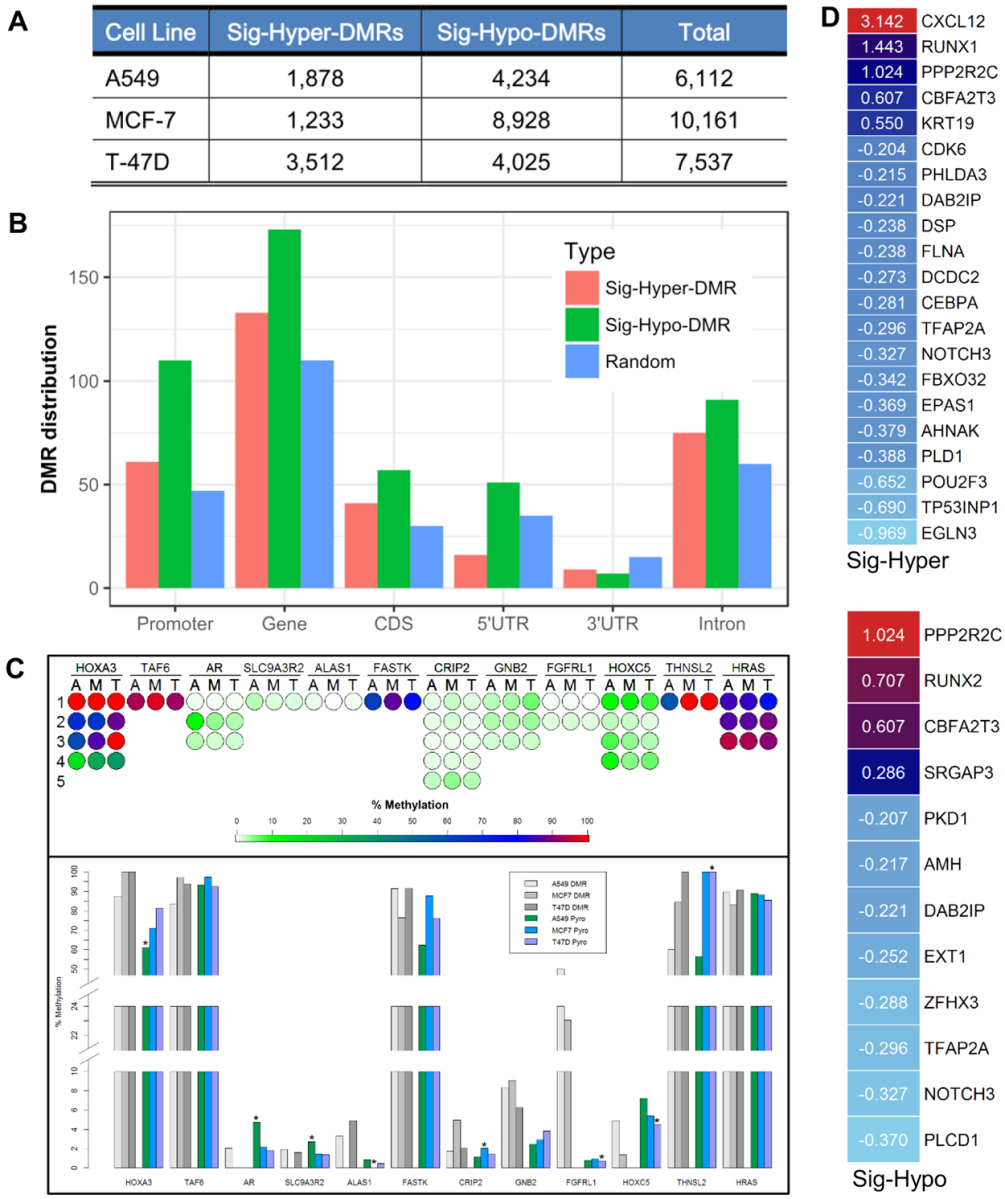
Genome-wide interrogation of significant differentially-methylated CpG sites and regions across 19 cell types. (**A**) The significantly hyper- and hypo-DMRs identified from three benchmark cell types, A549, MCF-7 and T-47D; (**B**) The identified TSGs’ components overlapping with Sig-Hyper-DMR and Sig-Hypo-DMR in T-47D; (**C**) Upper panel: DNA methylation pattern of 12 genes in the first 1~5 CpG sites has been detected by pyrosequencing (A: A549; M: MCF7; T: T47D). Lower: average of pyrosequencing detected %Methylation of 1~5 CpG sites for 12 genes in the three cell lines (A549 Pyro: green, MCF7 Pyro: sky blue, T47D Pyro: dark purple) and their DMRs (A549 DMR: light gray, MCF7 DMR: medium gray, T47D DMR: deep gray). Asterisk represents the results as algorithm expected; (**D**) The differential expression status for the investigated TSGs using RNA-seq data (left panel: Sig-Hyper-DMRs; right panel: Sig-Hypo-DMRs).

We further categorized all statistically significant DMCs into DMRs with the published toolkits^26–29^. We preprocessed by methylKit with the thresholds: region’s mean methylation difference cutoff ≥ 20% (adjusted q-value ≤ 0.01), and we obtained 16,277 DMR candidates. Of all the identified DMRs in T-47D cells, 8,936 are hyper-methylated (Hyper-DMR) and 7,341 are hypo-methylated (Hypo-DMR). With the more stringent adjusted q-value ≤ 0.001 and differentially-methylated CpG base count ≥ 5 to define DMR, we detected 7,537 statistically significant DMRs (Sig-DMR), 3,512 significant hyper-DMRs (Sig-Hyper-DMR) and 4,025 significant hypo-DMRs (Sig-Hypo-DMR). We also detected MCF-7 and A549 cell type specific DMRs respectively (Fig. 3A).

We associated those T-47D specific DMRs with ~1,200 currently known tumor suppressor genes^18^, by overlapping each DMR with TSGs’ genomic regions, promoter, 5′UTR, 3′UTR, CDS, and intron. We found 133 TSGs overlapping with the Sig-Hyper-DMRs and 173 TSGs with Sig-Hypo-DMRs, respectively (Fig. 3B).

Together we experimentally validated the cell type-specific DMRs in the selected three cell types, A549, T-47D and MCF-7 with a pyrosequencing assay (Fig. 3C). Our results illustrated that DNA methylation of the first 1~5 CpG sites in the randomly selected 12 genes has been detected (A: A549; M: MCF-7; T: T-47D, Fig. 3B - upper panel). An average of methylation of 1~5 CpG sites for eight genes showed their cell type-specific methylation patterns in A549 (red), M-CF7 (blue) and T-47D (green) (Fig. 3C - lower panel), respectively. The other four genes only showed cell type-specific methylation at individual CpG sites.

Furthermore, we examined their differential expression levels in T-47D with estrogen (E2) treatment vs. control (DMSO) samples using RNA-seq data from ENCODE, and found many genes, especially the corresponding TSGs, showed significantly differentially expressed (absolute log2 fold change ≥ 0.2, adjusted p-value ≤ 0.05; Fig. 3D, upper panel depicting the differential expression of TSGs in Sig-Hyper-DMRs and lower panel for TSGs in Sig-Hypo-DMRs**)**. The Supplemental Material Figure S4 presents the genome-wide DMC distribution and related statistical properties.

### Interrogating genomic region-dependent DNA methylation patterns

During cancer development and progression, changes in DNA methylation occur mostly within gene promoter (2,000 bp centered on TSS), CpG island and CpG island shore (1,000 bp centered around CpG island)^11, 30^. Thus, we particularly examined six genomic regions to understand genomic region-dependent DNA methylation patterns. In addition, we were only focused on these regions with TF binding. Region I – CGI.NP: CpG islands outside promoter regions, II – CGIS. NP: CpG island shores outside promoter regions, III – P.CGI: CpG islands within promoter regions, IV – P.CGIS: CpG island shores within promoter regions, V – P.NCGI: promoter regions out of CpG islands and VI – P.NCGIS: promoter regions out of CpG island shores, illustrated in the Supplemental Material Figure S5(I).

**Figure 4:**
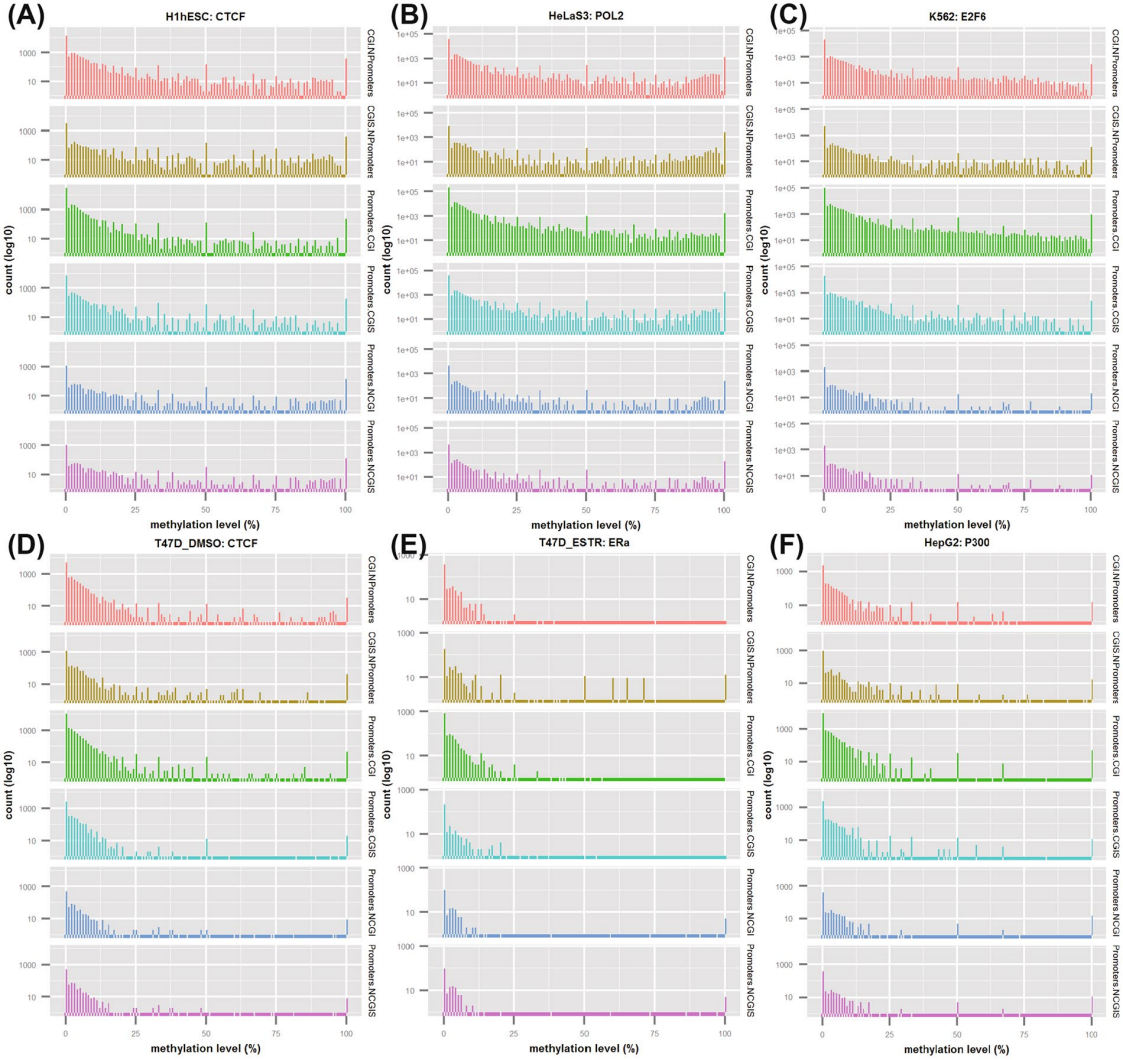
Interrogation of cell type-specific and genomic region-dependent DNA methylation distribution among six genomic regions across five cell types, namely H1-hESC, HeLa-S3, K562, T-47D (DMSO), T-47D (ESTR) and HepG2, within the cell-type and TF combinations. X-axis depicts the methylation level distribution (0~100%) and y-axis for the CpG loci (sites) count at each specific methylation level.

We observed there exists two distinct region-dependent patterns. One is hyper-methylation in Regions I, II, III and even in IV, but semi-hyper-methylation in Regions V and VI (Fig. 4A–C), and the other is hypo-methylation in Regions I, II, III, and IV, and even low methylation in Regions V and VI (Fig. 4D–F). The results indicated that DNA methylation in CpG islands and their shores exerted a cell type specificity than those in promoters since Regions V and VI (the promoter sub-regions independent of CpG islands and their shores) generally showed hypo-methylated. These distribution patterns were recapitulated in many TFs from other cell types (Supplemental Material Figure S5).

We speculated that such features, especially TFs’ binding at the hypo-methylated regions, might be critical for studying transcriptional regulation in cancer cells, such as ERα and CTCF in T-47D cell, as hypo-methylation triggers targeted promoter activity and its downstream transcriptional regulation^8, 31^. Indeed, we also found the CpG island-related sub-regions (independent of promoters) were overlapped with several sets of known enhancers. This is in line with other previous findings that enhancers, rather than promoters, are embedded comparatively more various DMRs in the context of a specific developmental and disease stage^32, 33^. It is noted that in addition to enhancer regions there might be other non-coding regions within the CGI-related sub-regions that might be important for transcriptional activities.

### Statistically integrating DNA methylation and gene expression

**Figure 5:**
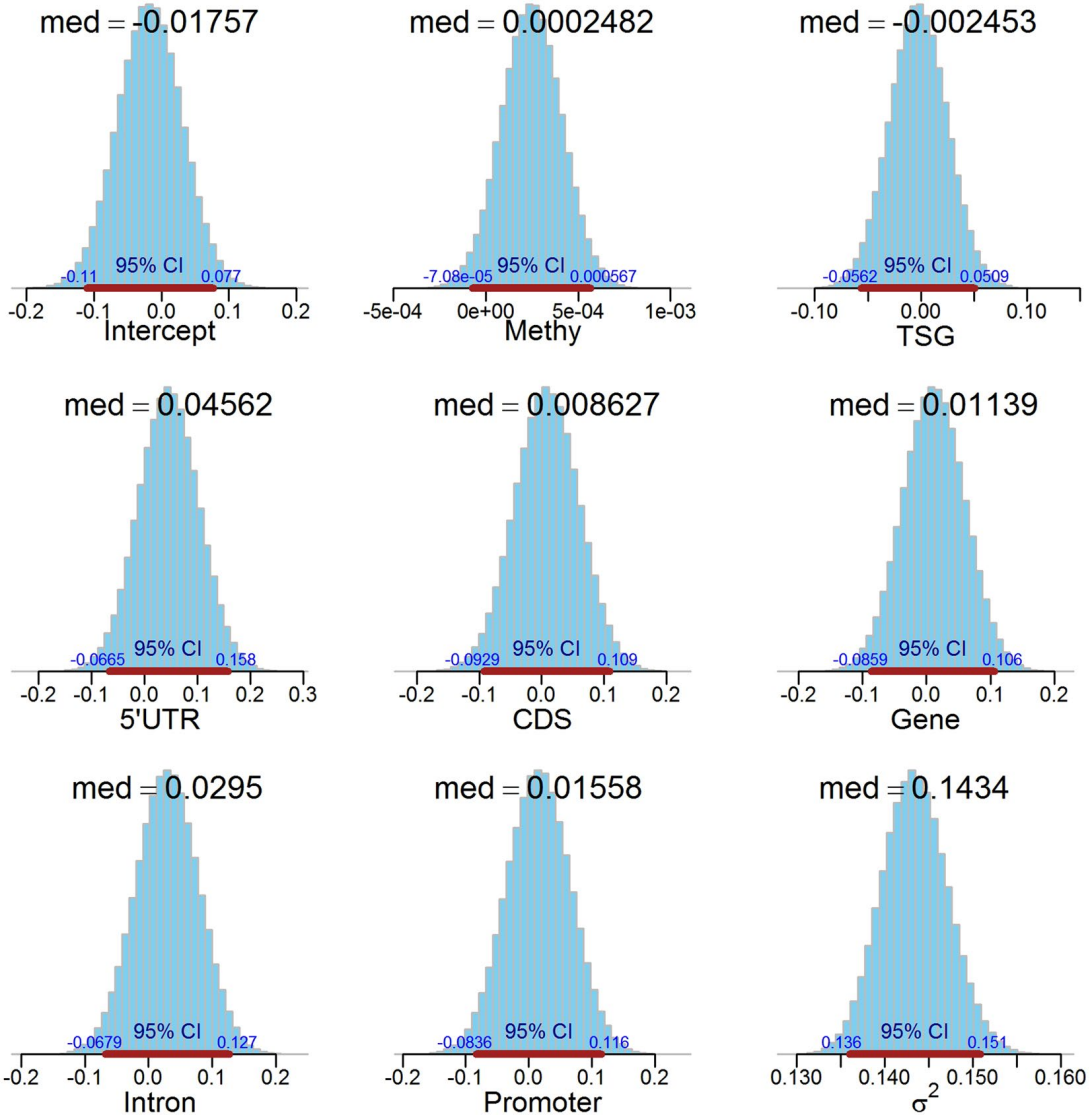
Statistics for Gaussian regression for differential expression (log2 fold change from RNA-seq) based on methylation level, TSG status, and DMR annotation region distribution (gene body, intron, promoter, 5′UTR, 3′UTR and CDS). Each plot lists the median (med) for sampling distribution, together with 95% confidence interval (CI) and start/end positions.

Next, we proposed a Bayesian regression model using Markov Chain Monte Carlo method to characterize the underlying association among DNA methylation, genomic segment distribution, differential gene expression level and tumor suppressor gene status (See the Method section for the details). The MCMC sampling distributions for modeling were given in Fig. 5, with the trace from genomic segment 3′UTR omitted automatically due to the insufficient sample size acquired, 25 against total 1,590 for the hyper case, and 38 against 1,291 for the hypo case, respectively. The average sample size for the other five genomic segments is 563. Figure 5 lists the regression coefficient median (med) by MCMC sampling, together with 95% confidence interval (CI) indicated by a brown line below, with the range at each end.

To statistically validate the derived model, we further introduced other regression item, HPRO (hyper- or hypo-methylation), into the model; and compared both log marginal likelihood and selection possibility for the models using Bayes Factor method. We found that Equation 2 is statistically fit for the current data with much higher selection possibility, 0.9855578 against 0.0144422 for the model with another item HPRO introduced, although the log marginal likelihoods are similar for two models, −1335.051 for the initially proposed model and −1339.274 for the model with a new item. This difference is due to duplicate effects from the variants Methy and HPRO. Methy takes positive or negative differential methylation value, which has quantitatively reflected binary hyper- or hypo-methylation status, HPRO.

Thus, the model in Equation 2 can best depict the quantitative relationship of differential gene expression level for TSG with DNA methylation, and genomic segment distribution. The Supplemental Material Figures S6–S8 illustrated the hyper-, hypo-methylation cases, and the case for the model with a new variant HPRO introduced.

### Unraveling the interplay between DNA methylation and TF binding

**Figure 6:**
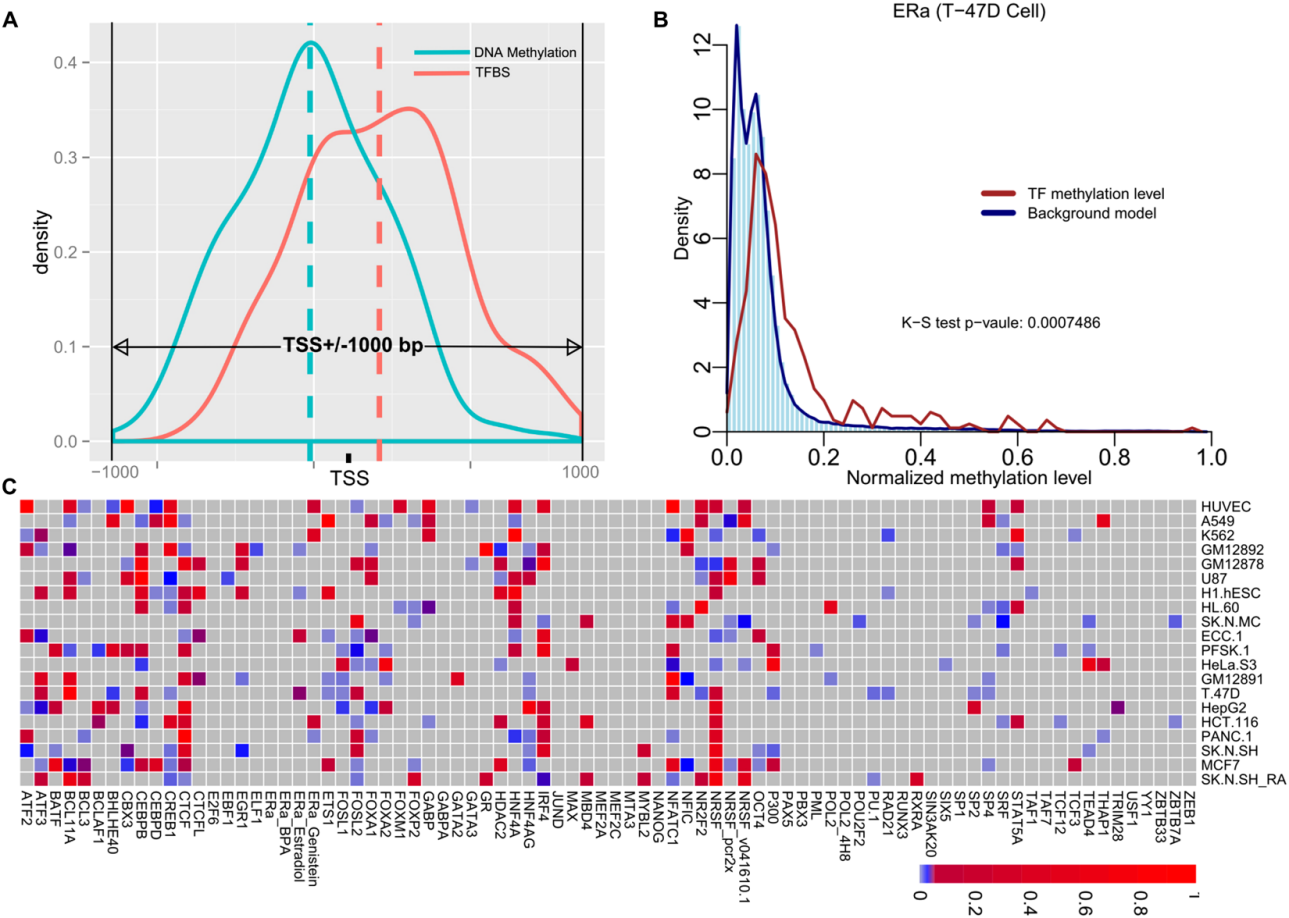
Genome-wide integration of DNA methylation and 82 TF binding from 19 cell types. (**A**) Schematic illustration in detecting differential DNA methylation status at TFBS regions (TSS ± 1000 bp) by Kolmogorov-Smirnov test; (**B**) Illustrative example for ERα in T-47D cell, blue dashed curve and histogram represent the background model in density distribution plot, the brown curve denotes the density plot for DNA methylation distribution at ERα binding site with range from 0 to 1; (**C**) A heatmap showing the differentially-methylated TFs, where vertical axis lists the 19 cell types and horizontal axis for the 82 TFs, 157 highly-differentiated entries (K-S test against the background model, BH-adjusted p-value ≤ 0.05, light blue to blue color), 156 lowly-differentiated entries (K-S test against the background model, BH-adjusted p-value > 0.05, red color), and 1,327 null entries (gray color).

To determine the quantitative interplay between DNA methylation and 82 TFs binding across the 19 cell types (Fig. 6), we introduced a Beta-distribution model for fitting the background methylation profile of each of 82 TFs in regions of a 2,000 bp length centered on TSS (TSS ± 1000 bp).

We then utilized a Kolmogorov-Smirnov (K-S) test in iteratively discriminating the background methylation status from TF-specific methylation pattern^21^ (Fig. 6A,B).

Based on K-S discriminative test analysis together with multiple testing correction, we identified 157 highly-differentiated methylation-TF (BH-adjusted p-value ≤ 0.05), 156 lowly-differentiated methylation-TF, namely the lowly-differentiated methylation-TF (BH-adjusted p-value > 0.05), and 1,327 un-differentiated methylation-TF (gray block), see Fig. 6C.

Our results showed many TF binding patterns are irrespective to their methylation status, while some key TFs prefer binding at lowly-methylated promoters. For example, POL2 (RNA polymerase II) showing highly-differential methylation among 10 cell types is consistent with its role as a form of eukaryotic RNA polymerase II, recruiting other core factors to the promoters of protein-coding genes during transcription initiation. ERα (estrogen receptor α) is highly differentially-methylated in ECC-1 cell; CEBP (an enhancer-binding protein) and TCF12 are found to be highly differentiated in MCF-7 cell, and interestingly, P300 (a transcription coactivator) and CTCF (an insulator) are also identified as highly differentially-methylated entries^34–37^. Our results suggest a diversified interplay mechanism between DNA methylation and TF binding activity across cell types.

## Material and Methods

### DNA methylation RRBS and ChIP-seq data sources

We obtained reduced representation bisulfite sequencing (RRBS) data and ChIP-seq data of 82 TFs in 19 cell types from the ENCODE Consortium Project^1^, which cover major human cancer cell types, human blood B-lymphocyte and embryonic stem cell types (Supplemental Material Table S1). We also retrieved Illumina Infinium Methylation Beadchip 450 K data, a CpG-specific array technology profiling over 450,000 CpGs covering 99% of all RefSeq genes, to perform comparison and correlation analysis with the RRBS data^23, 38, 39^.

### Definition of cell type-specific differentially-methylated CpGs and regions

We inferred the cell type-specific DMRs with a reference to a particular cell type based on the following definition:

1 $$\widehat{DM{R}_{i}}=DM{R}_{i}\backslash \mathop{\cup }\limits_{j\in S\backslash i}DM{R}_{j}$$

where *DMR*_*i*_ denotes the DMR set identified for the *i*-th cell type from the 19 cell type set *S*, $\mathop{\cup }\limits_{j\in S\backslash i}DM{R}_{j}$ denotes the DMR set union by all the other cell types from *S* except for the *i*-th cell type, the symbol\denotes the set deduction operation, and $\widehat{DM{R}_{i}}$ indicates cell type-specific DMR set for the *i*-th cell type after set subtraction from the other DMR sets.

### Biological validation experiments with pyrosequencing assay

Genomic DNA of MCF-7, T-47D and A549 cell lines were extracted with QIAamp DNA Mini Kit (QIAGEN) respectively, then bisulfite-converted with EZ DNA Methylation-Gold Kit (ZYMO RESEARCH). The DNA sequences of 12 cell type-specific DMRs were obtained from human genome, then input to Qiagen PyroMark Assay Design 2.0 software. PCR primers and pyrosequencing primers for the pyrosequencing experiments were generated by the software. Genes were amplified with primers listed in Table 1 using the converted genomic DNA as template.

**Table 1:** Primers used for pyrosequencing experiment.

Gene	PCR Forward	PCR Reverse	Pyrosequencing Primer	Biotin-labelled
HOXA3	GTTTAGGGAAGGGTTGGTTTAG	AACAATCCAACTCCTAATCTCCTCC	GGGTGATTTTTTTTAGTTTAGT	Reverse
TAF6	GGTTAAGGTGGTAGTTTGTT	CTAATCTTAAAACTAAAAACTCTTC	AACTCTTCCTACCCA	Forward
AR	AAGGAGGTGGGAAGGTAAG	ACTAACTCCACCCTTTTTCCCTCTATC	GTTGTATTTGTTTTTTATTTTTTAG	Reverse
SLC9A3R2	TAGAGTAGGGGAGAGATAGAGAGGTT	ACCAAACCCCTACCT	CCAAACCCCTACCTC	Forward
ALAS1	TAGGATGAGGTAGGGAAAAGAGATT	AAAAAACCAAACAAAAAAACCCACTTCTTA	AGGAGAGTAGGGGAATTT	Reverse
FASTK	AATGGAAGAGGAGGGGGATTTAGT	ACCCCCCCATAAAAAATAAATAATTTACAC	TTGTTTTTAGTTTTAAAATTGAGAT	Reverse
CRIP2	GTTATGGAAATAGAGTAATAAAGGGAAG	AACCCTAATAACTTAAACCTAAAAATCC	GGAGTTTGAGATTTTTTT	Reverse
GNB2	GTGGGAGAGGTTGAGGAAATGTT	CCACCCCCCTCACCAAA	AAAACCAAACTAAAAAAACCTAAAC	Forward
FGFRL1	GGGTTAGGGGTTTTAGTTGG	ACCCCCAAAACACACAACACTCAA	GGGGTTTTAGTTGGGTA	Reverse
HOXC5	TTTTATGAGAGAATTGGGTAAATATGG	TAACTTCTTATAACCAATCCAACTTA	ACTATAAATTTTCTACAAACAACC	Forward
THNSL2	ATGTGTTTAGGAGATTGGTGGTTTAGA	CCCCTAATACTAACATACCACATAATCCT	GTGTGTGTTTTGGTTT	Reverse
HRAS	AGAGTTTAGGGTTGGATAGGT	CTCCCAAACCTCTATAAACCCTTATC	GTTTGAGGTTTAGATATATTTTATG	Reverse

The PCR products were verified for size and quality with agarose gel electrophoresis. Biotin-labelled forward or reverse primers were used for PCR and the products were pulled down with streptavidin-coated agarose beads. The biotinylated single-strand DNA fragments were purified with PyroMark Q96 Vacuum Workstation (QIAGEN). The pyrosequencing was performed with pyrosequencing primers listed in Table 1 using PyroMark Q96 MD (QIAGEN). The sequencing results were analyzed with PyroMark CpG software (QIAGEN) to get the percentage of methylation level (%Methylation).

### Statistical modeling the association between DNA methylation and gene expression

We developed a Bayesian regression model with Markov Chain Monte Carlo sampling method to characterize the statistical association among differential gene expression (DGE) level (log2 fold change) acquired from the control and treatment samples by RNA-seq, methylation level (Methy), TSG category, and methylation level regarding genomic segment (GS) distribution. The Bayesian regression model takes the following form,

2 $$\begin{array}{rcl}{{\rm{DGE}}}_{i} & = & X{\rm B}+{\Xi }_{i}\\  & = & \sum _{i=1,j=1}^{m,n}{x}_{i,j}{\beta }_{i,j}+{\varepsilon }_{i}\\  & = & \sum _{i=1}^{m}[{{\rm{Methy}}}_{i}{\beta }_{1,i}+{{\rm{TSG}}}_{i}{\beta }_{2,i}+{{\rm{GS}}}_{i}{\beta }_{3,i}]+{\varepsilon }_{i}\end{array}$$

where *i*∈*N*, DGE_*i*_ stands for log2 fold change values for *i*-th annotated gene, Methy_*i*_ stands for a mean methylation value (in percentage) of the *i*-th annotated gene’s hosted DMR, TSG_*i*_ for the status whether or not the *i-*th annotated gene belongs to TSG (binary TRUE or FALSE), GS_*i*_ for the *i*-th gene’s annotated genomic segment base (promoter, 5′UTR, gene body, introns and 3′UTR), and the error entry follows Gaussian distribution, ${\varepsilon }_{i} \sim N(0,{\sigma }^{2})$.

We further assume standard semi-conjugate priors,

3 $$\begin{array}{c}\beta  \sim N({b}_{0},{B}_{0}^{-1})\\ {\sigma }^{-2} \sim Gamma({c}_{0}/2,{d}_{0}/2)\end{array}$$

where *β* and σ^−2^ are assumed a priori independent, *b*_0_, ${B}_{0}^{-1}$, *c*_0_ and *d*_0_ are the initial values predefined for Gaussian and Gamma distribution, respectively.

Bayes Factor analysis was adopted to compare the model candidates and perform model selection using log marginal likelihood and selection possibility. Then the posterior information of regression model with Gaussian errors is acquired using Gibbs sampling^40–43^. We sampled 500,000 iterations with the first 1,000 times truncated to ensure trace convergence.

### Detecting differential DNA methylation status at promoter regions

To identify TFs with specific methylation status around their TSS regions, we adopted Kolmogorov-Smirnov (K-S) test for statistically differentiate the background model and each TF’s DNA methylation distribution. For each TF, its peak binding region for measuring methylation level is defined as a length of 2000 bp centered on TSS (TSS ± 1000 bp), mainly for covering each promoter region.

We selected the RRBS data for measuring each TF’s methylation status. With the aggregated DNA methylation level from the 82 TFs across all the 19 cell types, we constructed their methylation distribution background model with the normalization and fitting. Then we adopted the K-S test for determining the statistic p-value between TF’s methylation distribution density and the background model. We defined those entries with p-value ≤ 0.05 as the significantly differential methylated compared with background model.

### Tools used in the curation and analysis

Bowtie2^44^ was used to align sequencing reads, SAMtools^45^ and BAMtools^46^ were used to process the aligned sequencing reads, methylKit^29^ was used to analyze part of RRBS data, and DESeq^47^ was used to analyze RNA-seq data.

## Discussion

Integration of multi-platform and cross-cell-types omics information enables the thorough interrogation of genomic features with undiscovered biological functions. Till now, there is still limited systematic analysis for hundreds of data sets across different data types and multiple cell types.

Our work conducted the systems integration of 19 ENCODE cell types about the cell type-specific DNA methylation and its impacts on transcriptional regulation. The systematic analysis on DNA methylation within predefined genomic regions or segments revealed that DNA methylation in CpG islands and CpG islands shore characterizes a specific cell type’s methylation status, which may act as a hallmark in studying transcriptional regulation for the different cancer cells.

We found that promoter regions and CpG islands in T-47D tend to be hypo-methylated, with 44% and 75% of total significantly hypo-methylated DMCs, respectively while CpG islands shores only cover 9%. Annotation showed hyper- and hypo-methylated regions are embedded with 634 and 511 TSGs, respectively, strongly suggesting the mechanism and functionality underlying the TSGs tightly linked to DNA methylation. Future experiments may be needed to determine whether DNA methylation plays a casual role in hyper-methylation associated TSGs by switching methylation status to hypo-methylation.

Through the cross cell-type comparison of differential gene expression, DNA methylation, and differentially-methylated CpG sites and genomic regions, we derived a quantitative formula to measure their relationship using our proposed Bayesian regression model. Our Bayesian model reveals that methylation sites in 3′UTR have much less impact on transcriptional regulation than other regions.

In all, our systematic analysis reveals cell type-specific and genomic region-dependent regulatory patterns with a breast cancer cell T-47D as a benchmark cell type, and provides an efficient approach in integrating hundreds of various omics-seq data together.

### Availability

The analyzed intermediate results and tables for the project are deposited at: https://github.com/gladex/PanCanMAP.

## Electronic supplementary material

Suppl. Info

Suppl. Dataset
