# Supplementary material for: Integration of DNA methylation and gene transcription across nineteen cell types reveals cell type-specific and genomic region-dependent regulatory patterns
Source: Sci Rep. 2017 Jun 15;7:3626. doi: 10.1038/s41598-017-03837-z (PMC5472622; doi:10.1038/s41598-017-03837-z)
Supplement: Supplementary file 1 — Suppl. Info [file 41598_2017_3837_MOESM1_ESM.doc]

**Supplemental Materials**

**Integration of DNA methylation and gene transcription across nineteen cell types reveals cell type-specific and genomic region-dependent regulatory patterns**

Binhua Tang, Yufan Zhou, Chiou-Miin Wang, Tim H-M Huang and Victor X Jin

1. **Summary of collected data from the ENCODE Consortium Project.**

We collected ChIP-seq data for 82 transcription factors (TFs), reduced representation bisulfite sequencing (RRBS) and Illumina Infinium Methylation Beadchip 450K data for DNA methylation profiles in 19 cell lines from the ENCODE Consortium Project.

1. Breast cancer: MCF-7, T-47D;
2. Cervical cancer: HeLa-S3;
3. Endometrial cancer: ECC-1;
4. Blood cancer: HL-60, K562;
5. Brain cancer: SK-N-MC, SK-N-SH, SK-N-SH_RA, PFSK-1, U87;
6. Liver cancer: HepG2;
7. Colon cancer: HCT116;
8. Pancreas cancer: PANC-1;
9. Lung cancer: A549;
10. Human blood B-lymphocyte: GM12878, GM12891, GM12892;
11. Human embryonic stem cell: H1-hESC.

**Table S1** lists the statistics for the profiled CpG site count, site count with at least 10 mapped sequencing reads (10x enriched site), the percentage of the 10x enriched sites against the total sites, and total reads mapped to those profiled sites.

In our analysis, we select the CpG sites covered by at least 10 mapped sequencing reads as recommended by the ENCODE protocol.

The minimum percentage of the 10x enriched sites is 46.39% (GM12891), the maximum is 75.74% (PFSK-1), and the mean percentage is 63.85%. On average, each cell line is covered by 36,061,184 reads, with the minimum 12,661,734 (GM12891) and the maximum 59,680,360 (A549).

**Table S1**. Statistics for the 19 RRBS-profiled ENCODE cell lines


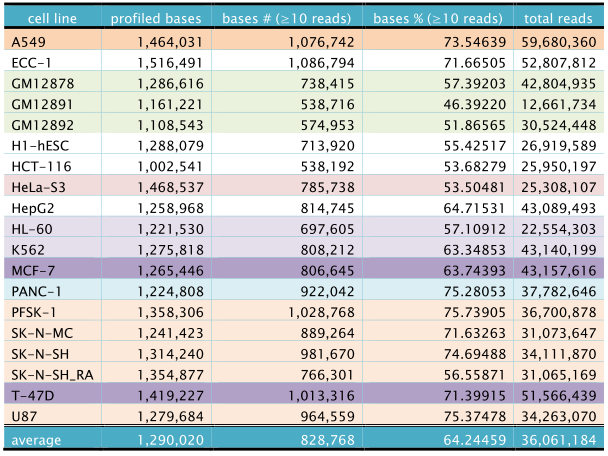


1. **Genome-wide DNA methylation profiling comparison between two platforms**

In **Figure S1**, although RRBS profiling tends to be enriched in highly methylated CpG level, we find all those cells have bimodal distributions of DNA methylation profile, except for K562 with unimodal distribution (for both 450K and RRBS platforms).

**
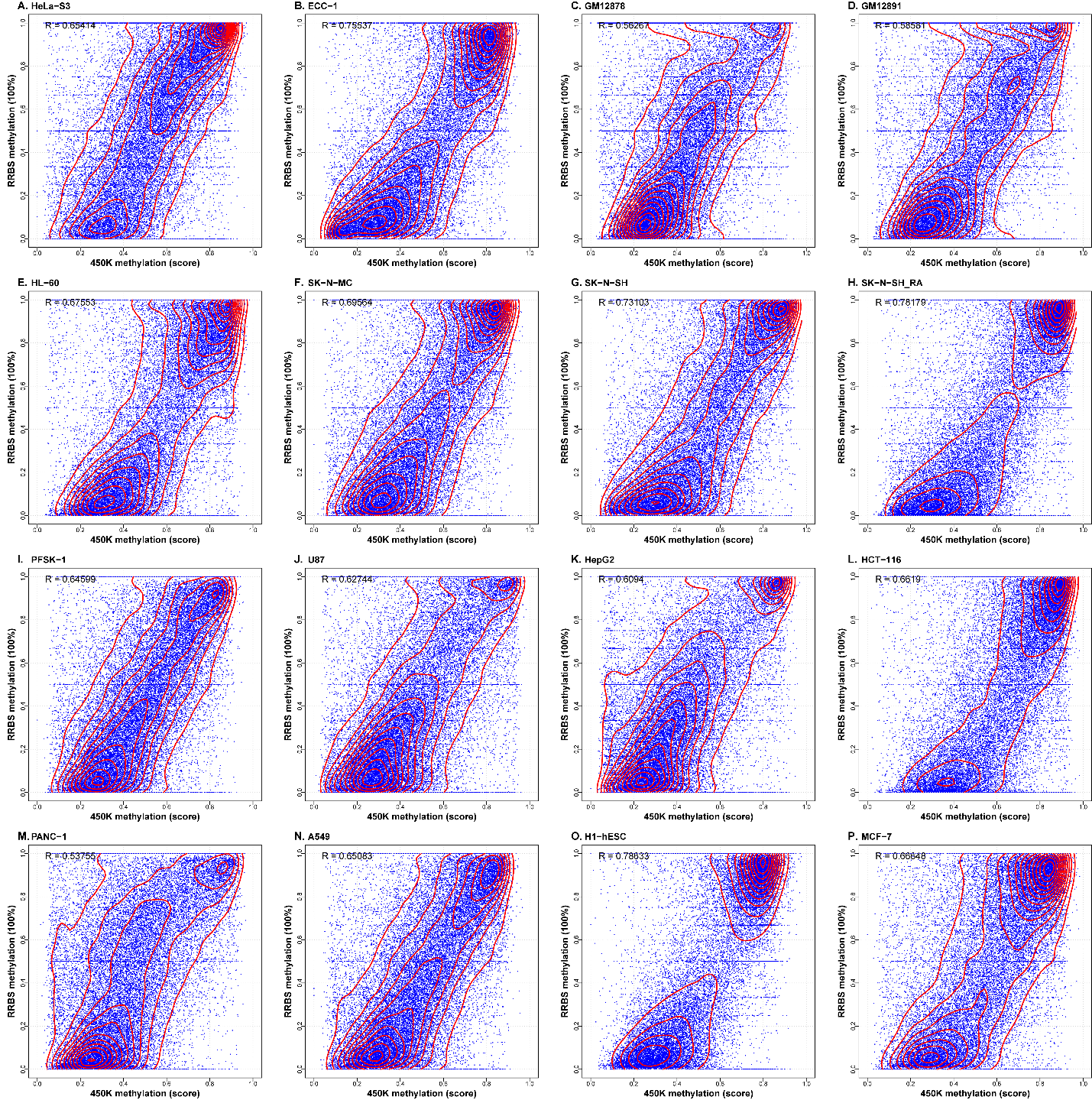
**

**(I) Pairwise comparison** (Illumina Infinium 450K vs. RRBS)


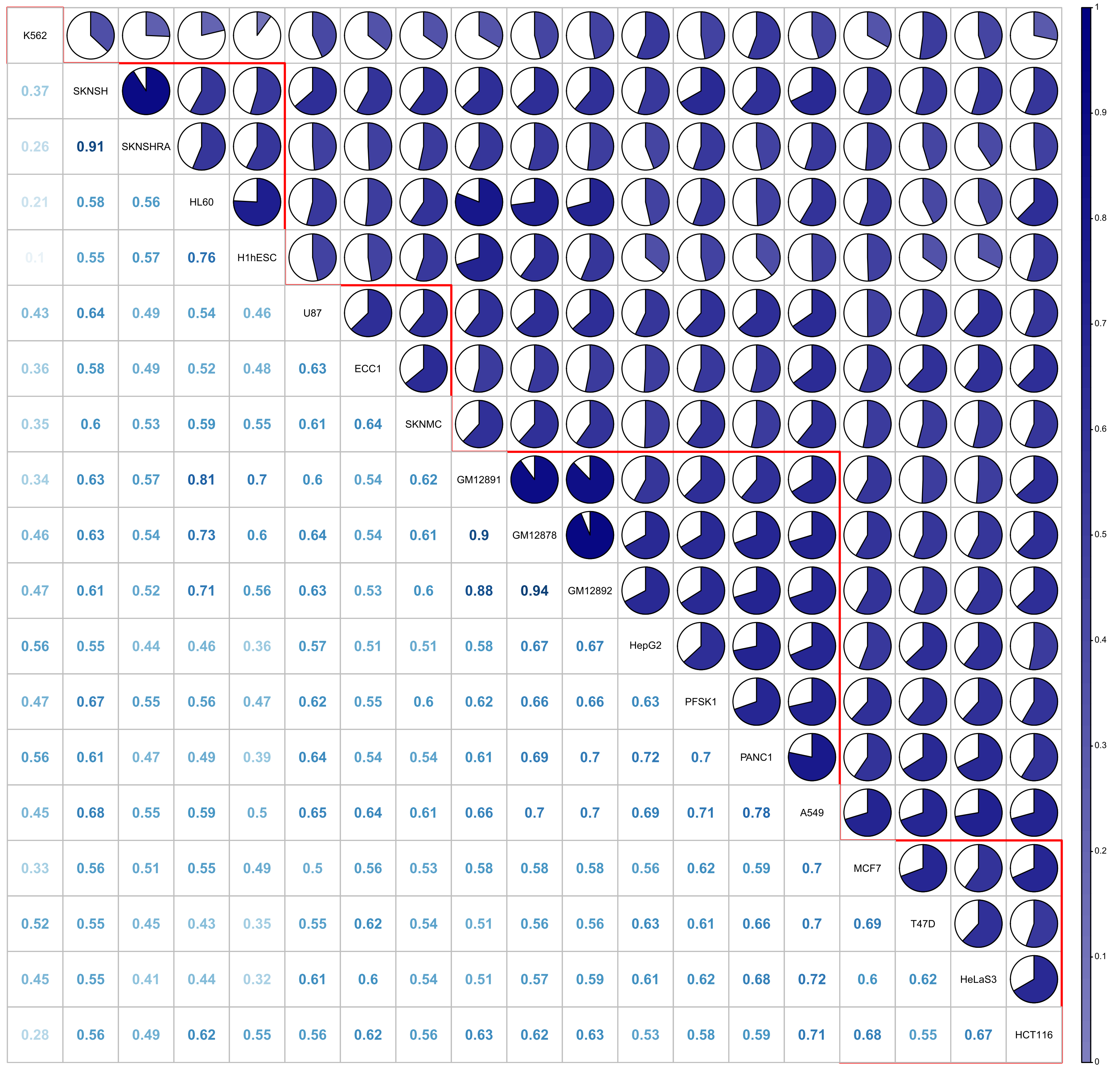


**(II) Illumina Infinium 450K platform**

**Figure S1.** **(I)** Genome-wide DNA methylation profiling comparison between two platforms (Illumina Infinium 450K and RRBS) for other cell types. *Pearson* correlation coefficient is given on the top left corner, respectively; **(II)** Genome-wide *Pearson* correlation chart for DNA methylation profiles across 19 cell types (Illumina Infinium 450K). Each diagonal entry gives cell type’s name and respective methylation percentage histogram; lower off-diagonal entry denotes genome-wide methylation level distribution; and upper entry gives the pairwise correlation among the 19 cell types.


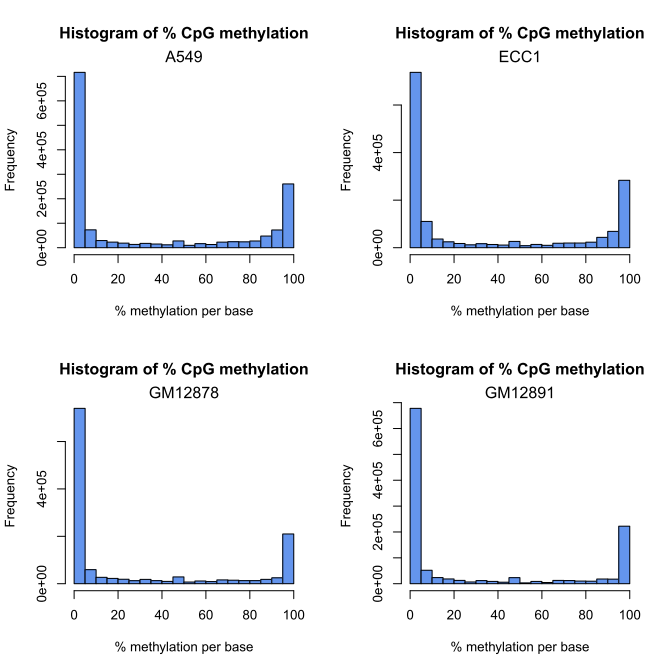

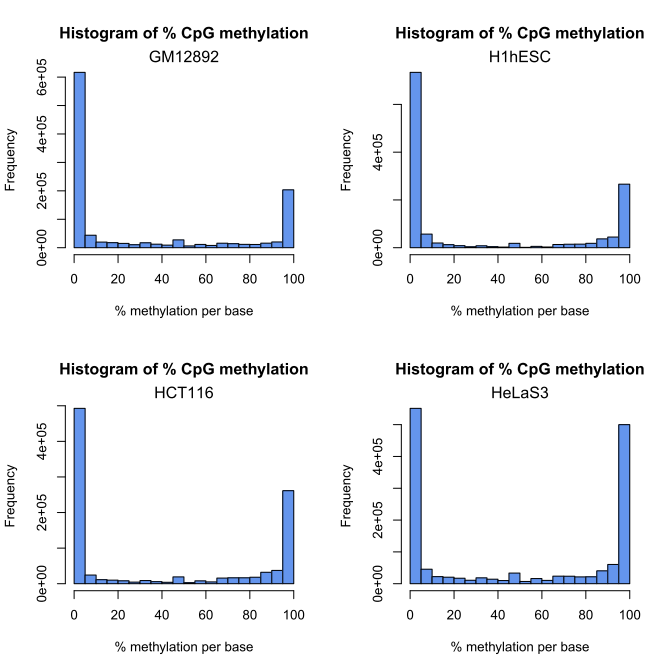


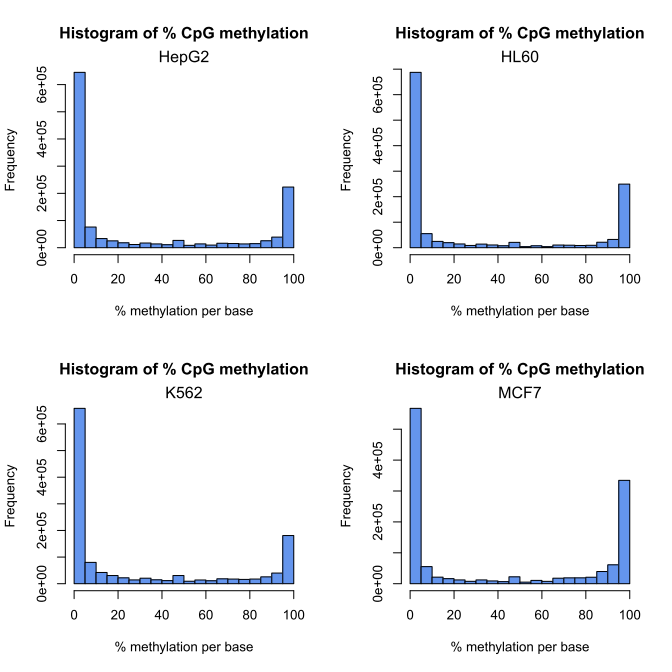

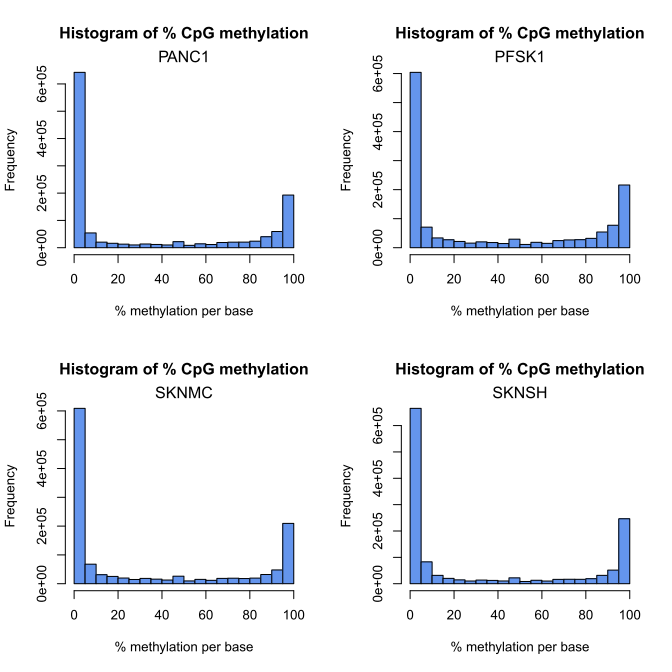


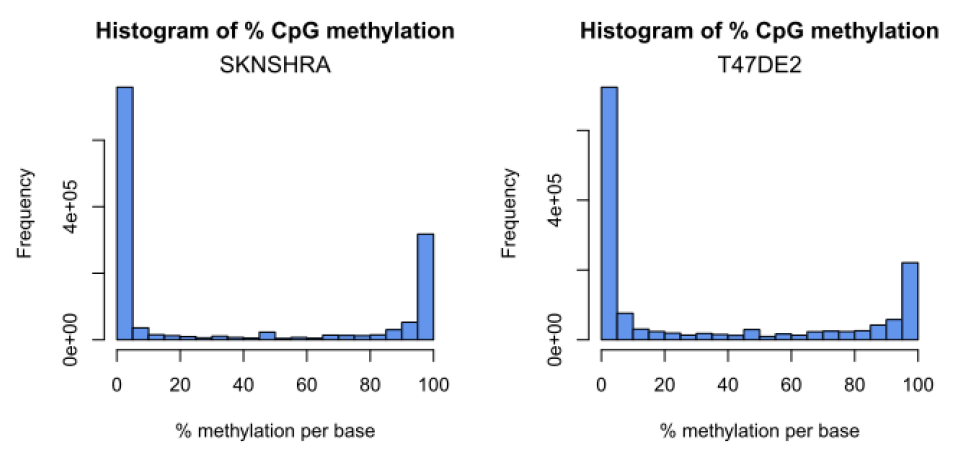

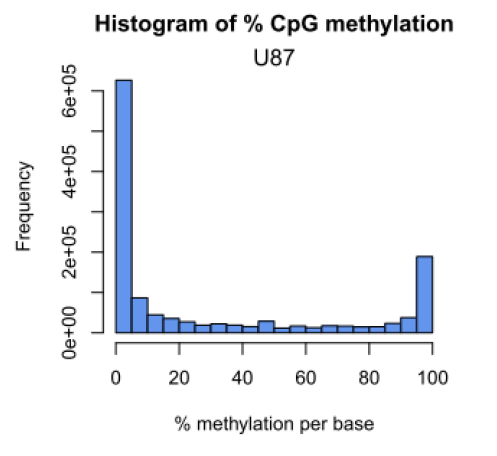


**(I) RRBS platform**

**
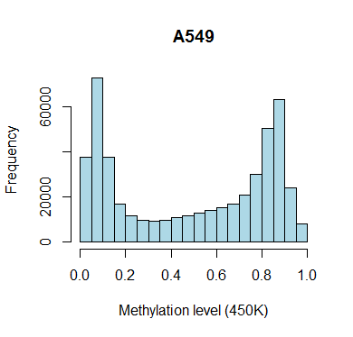

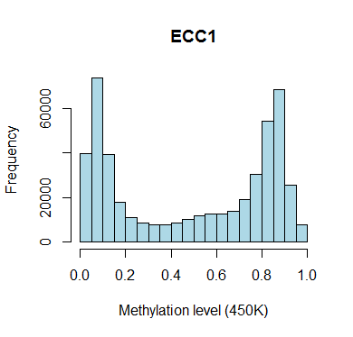

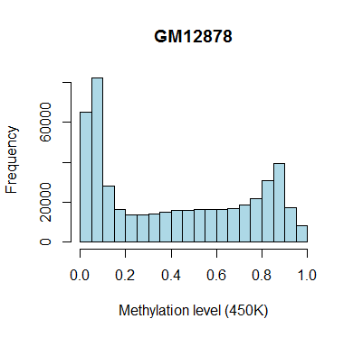

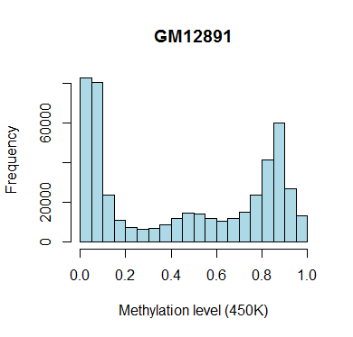
**

**
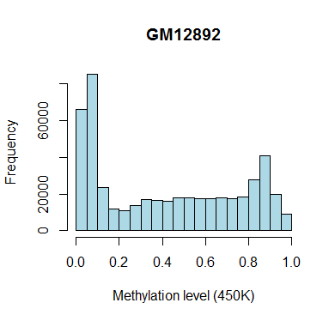
** **
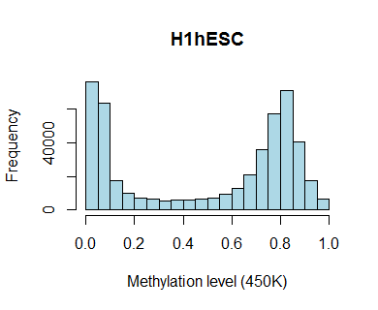

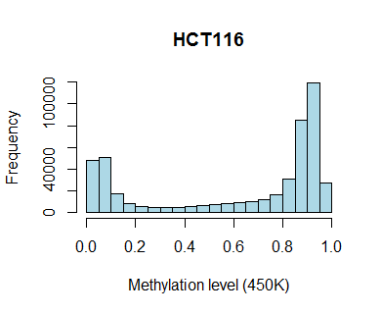

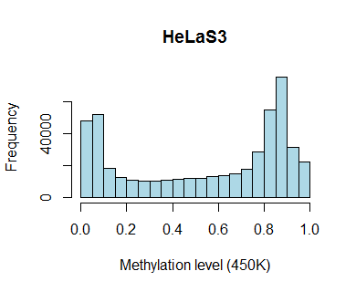
**

**
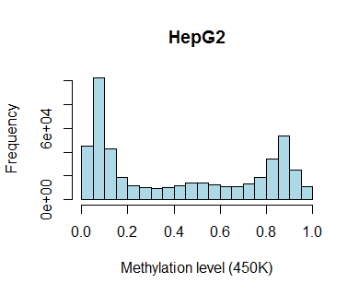
** **
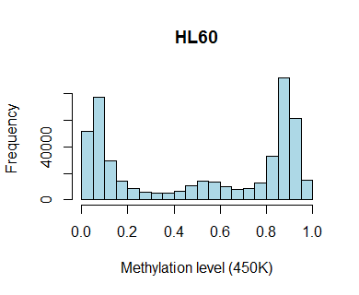

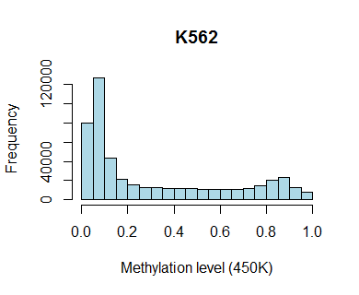

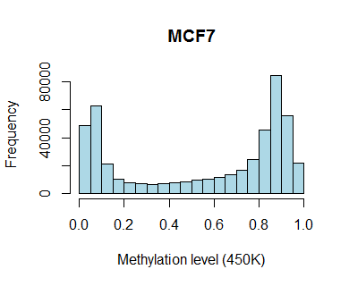
**

**
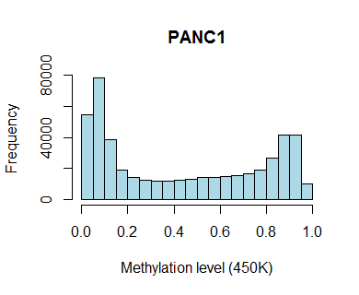
** **
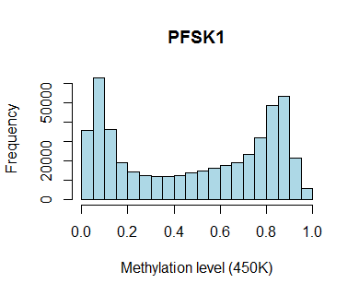

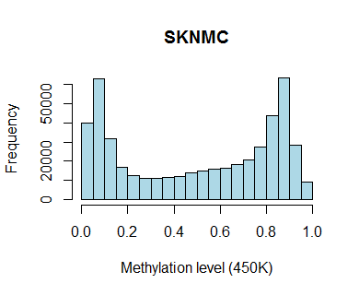

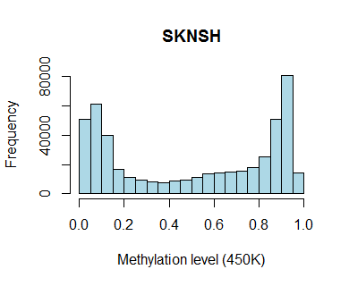
**

**
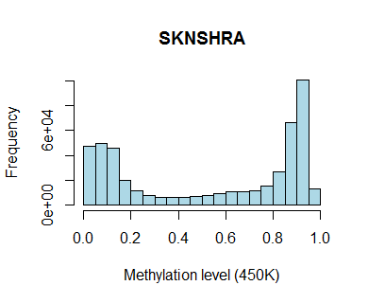

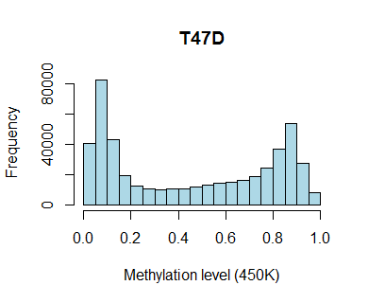

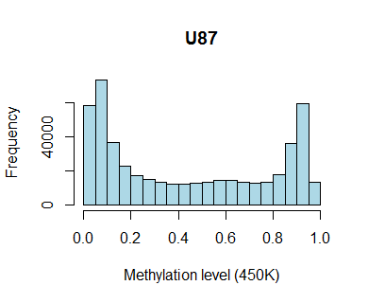
**

**(II) Illumina Infinium 450K platform**

**Figure S2.** Histogram of DNA methylation distribution for the 19 ENCODE cell types, and basically the profiling data follows bimodal distribution. (I) RRBS platform; (II) Illumina Infinium 450K platform.


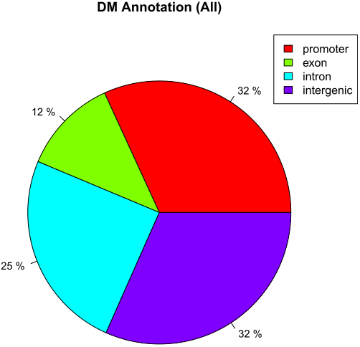

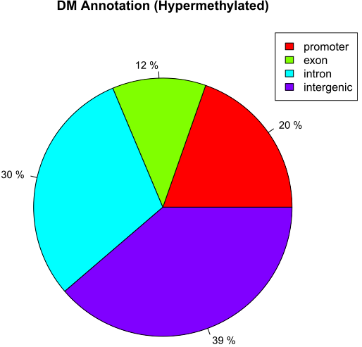

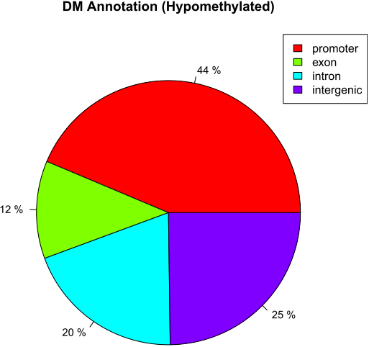

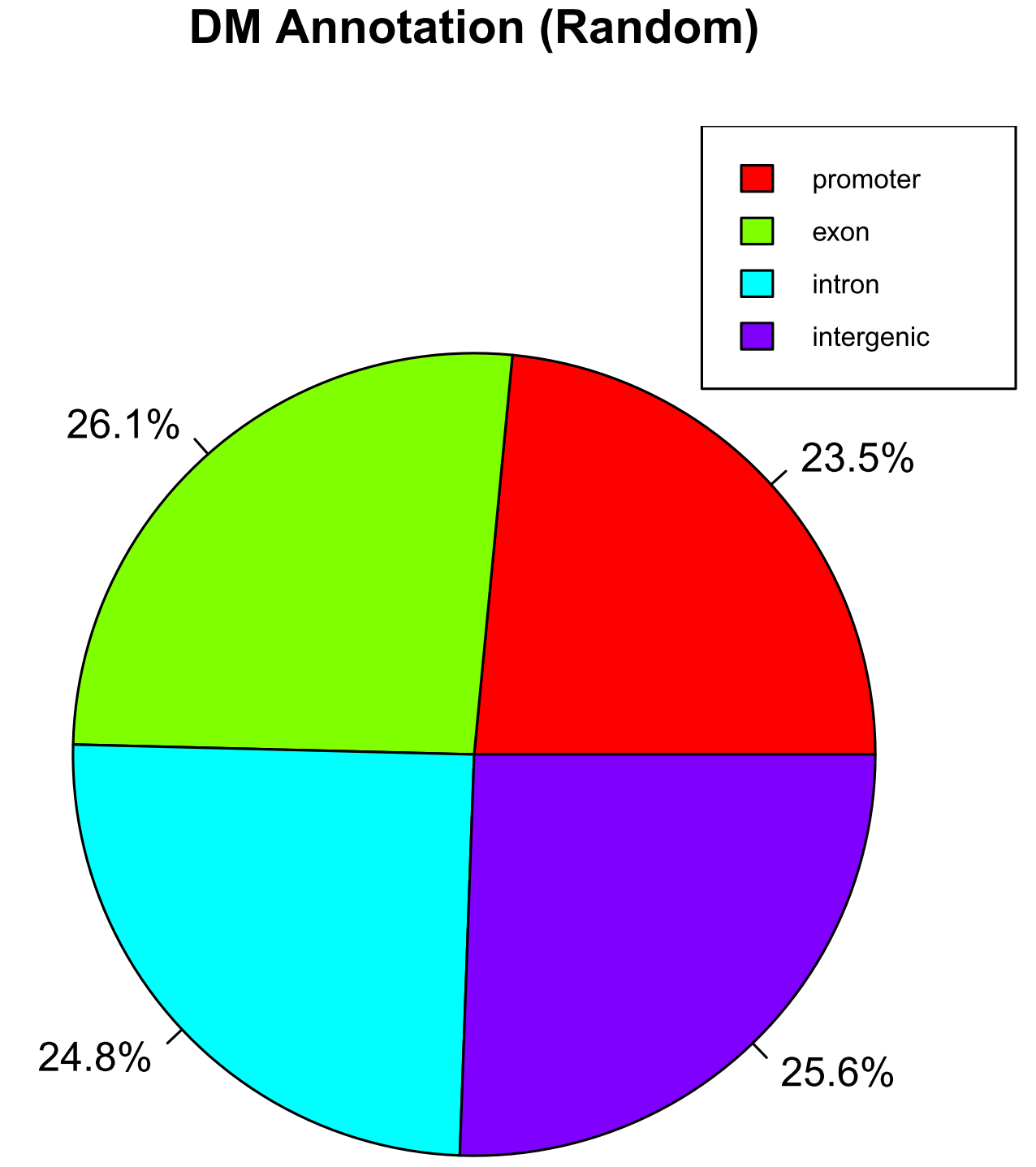


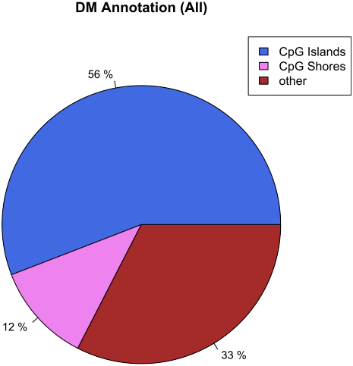

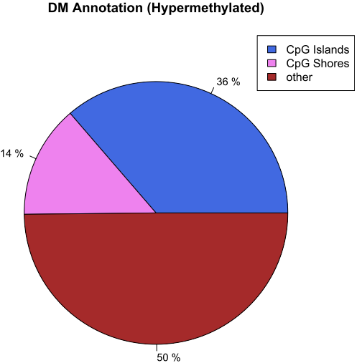

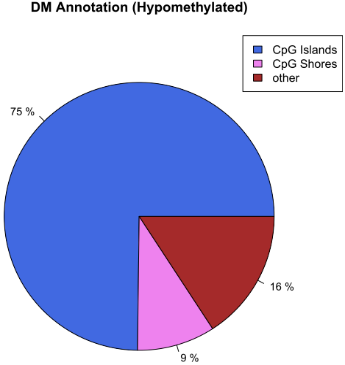

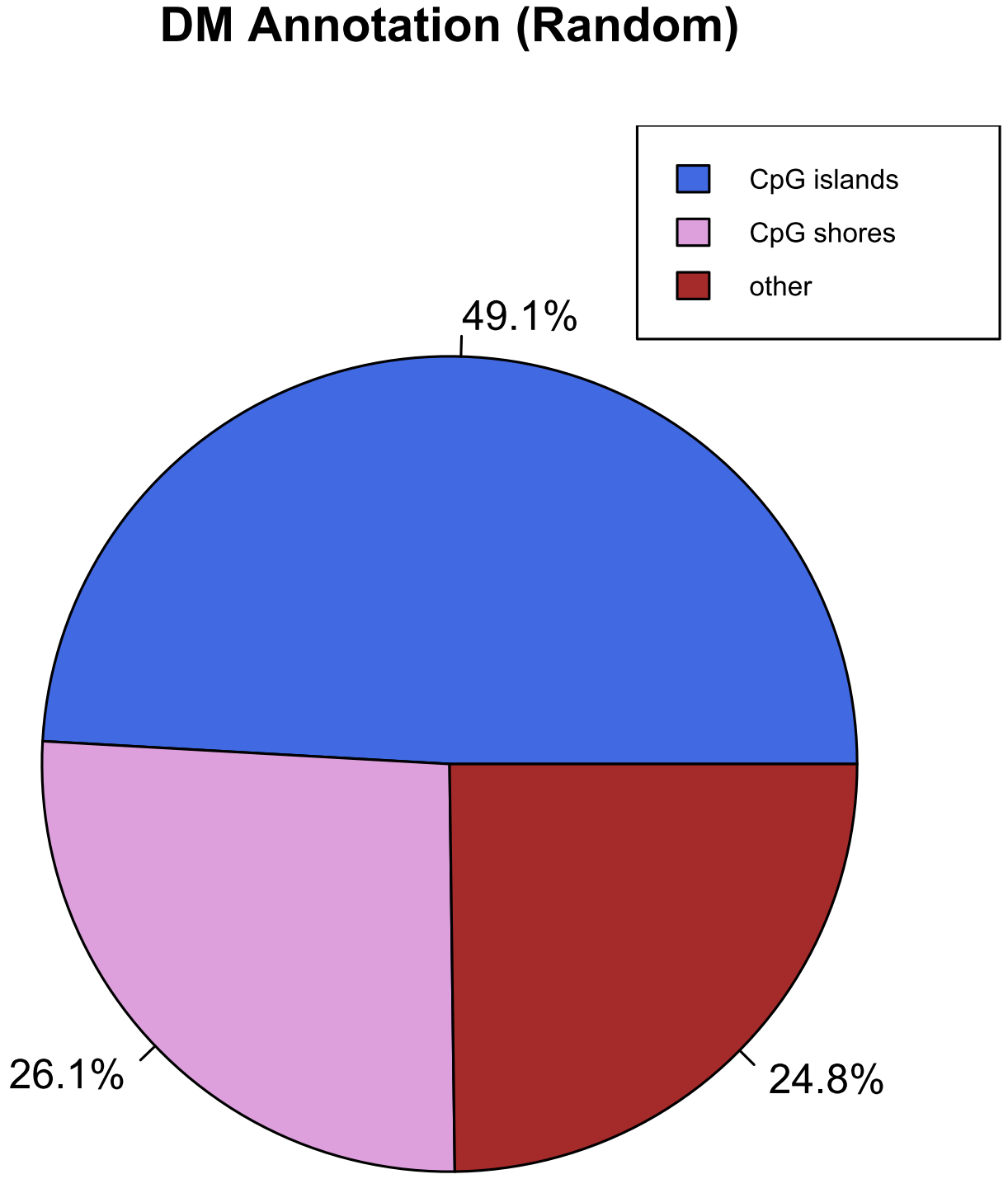


**Figure S3.** Statistical distribution for the annotated significantly DMCs (total count, hyper-methylated and hypo-methylated bases) in genomic regions (promoter, exon, intron, intergenic on the three upper subplots; CpG island, CpG shore and other regions on the three lower subplots).


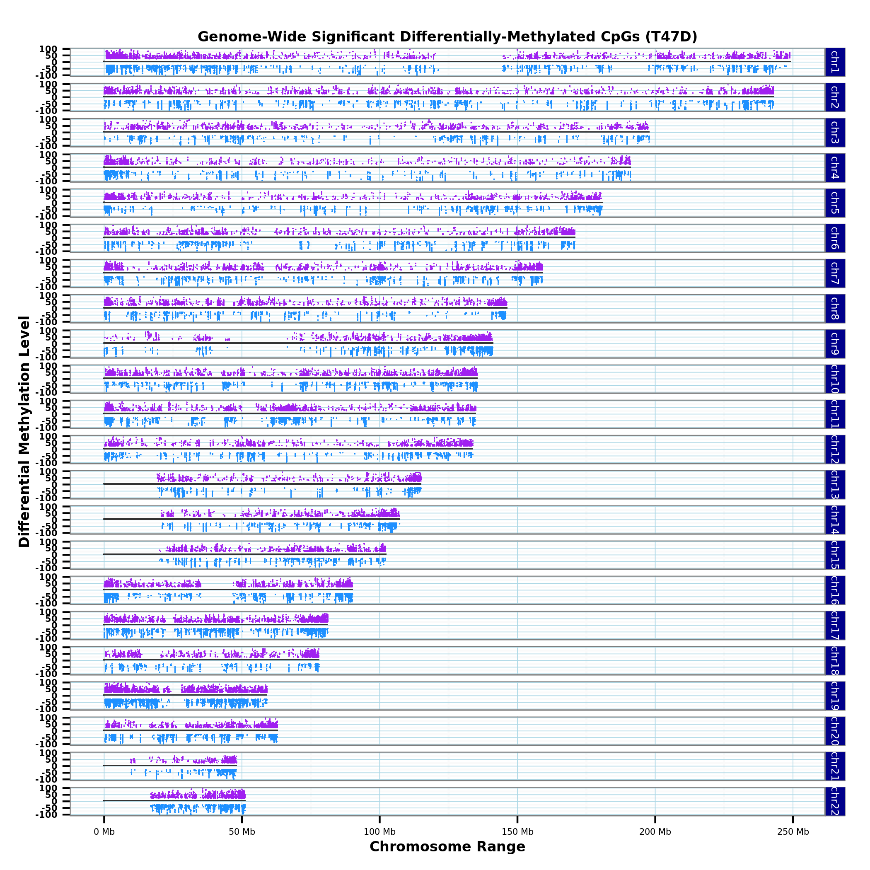

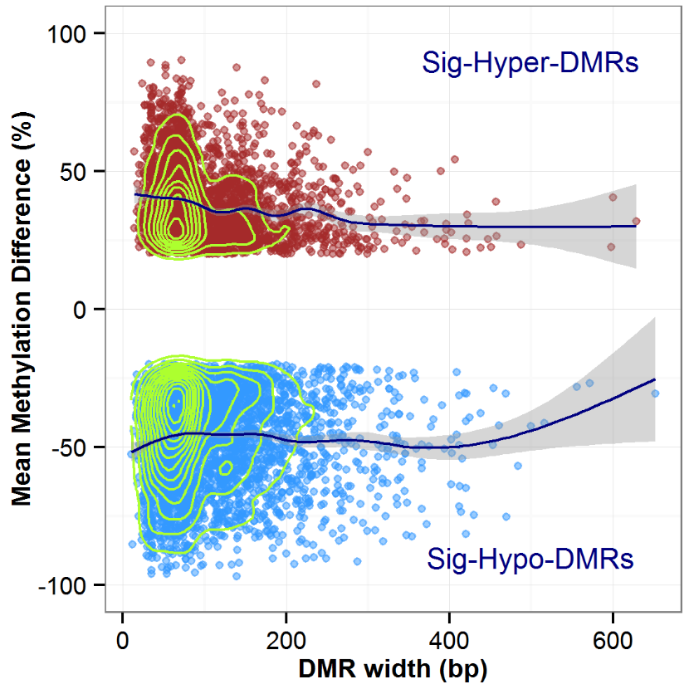


**(A) (B)**

**Figure S4**. (**A)** Chromosome-wise distribution for significantly differentially-methylated CpGs in T-47D, compared with all other cell types. (**B**) Within the 7,537 identified Sig-DMRs, the minimum DMR width is 10 bp, the maximum DMR width is 652 bp and the average width is 101 bp for all the identified DMRs. An anti-correlation between DMR width and its mean methylation difference, for both Sig-Hyper-DMR (top half) and Sig-Hyper-DMR (bottom half) pairs.

1. **Analysis on genomic region and DNA methylation pattern.**

We selected UCSC RefSeq Gene (HG19 coordinate) as the reference genome. The genomic segments, promoter, 5’UTR, CDS, gene body, intron and 3’UTR, follow the preprocess results from public toolkits, QDMR, BSmooth and methylKit, also mentioned in the Results section (main text).

For CpG island, we adopted the commonly-used definition and UCSC reference information for computational investigation, and there is online resource for retrieving the information, http://genomewiki.ucsc.edu/index.php/CPG_Islands; for CpG island shore, we define it as the region with 1,000 bp centered on CpG island. We mentioned the definition and related information in main text. Figure S5(I) illustrates the definition of the six regions for comparing DNA methylation patterns.

**
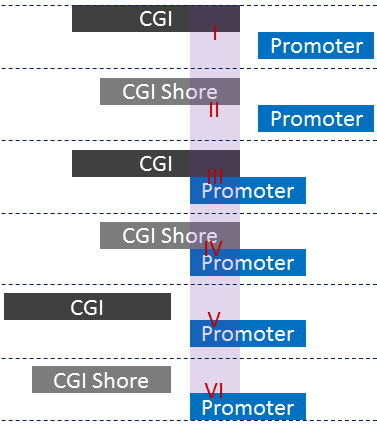
**

**(I)**


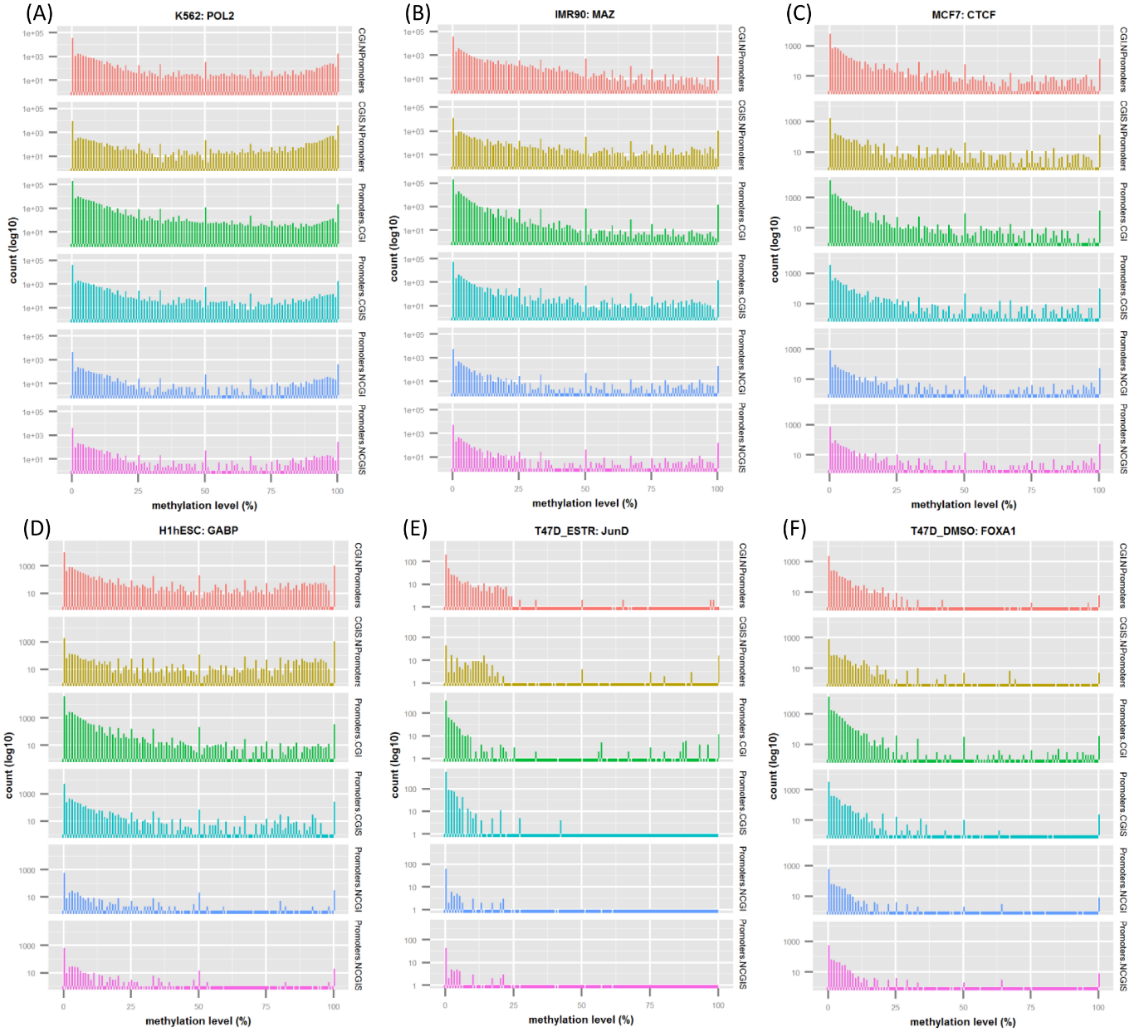


**(II)**

**Figure S5.** **(I)** Definition of six interconnected regions based on CpG island (CGI), CpG island shore (CGI Shore) and promoter; **(II)** Interrogation of cell-type-specific and regional-dependent DNA methylation distribution among six genomic regions across five cell types, namely K562, IMR90, MCF-7, H1-hESC, T-47D (ESTR) and T-47D (DMSO), within the cell-type and TF combinations.

1. **Bayesian regression analysis with MCMC sampling.**


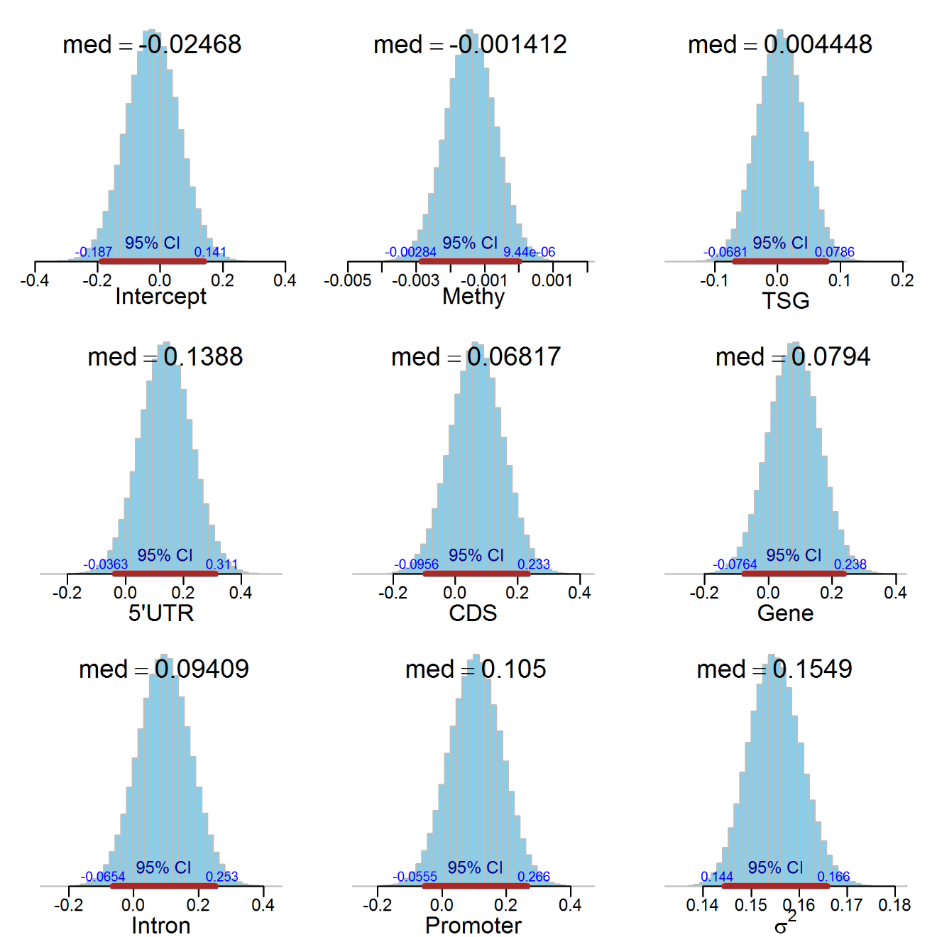


**Figure S6.** Statistics for Gaussian regression for differential gene expression (log2 fold change from RNA-seq) based on methylation level, gene category (tumor suppressor gene or not) and DMR annotation region distribution (gene body, intron, promoter, 5’UTR, 3’UTR and CDS). Each plot lists the median (med) for sampling distribution, together with 95% confidence interval (CI) and start/end positions. The analysis result is for hyper-methylation case.


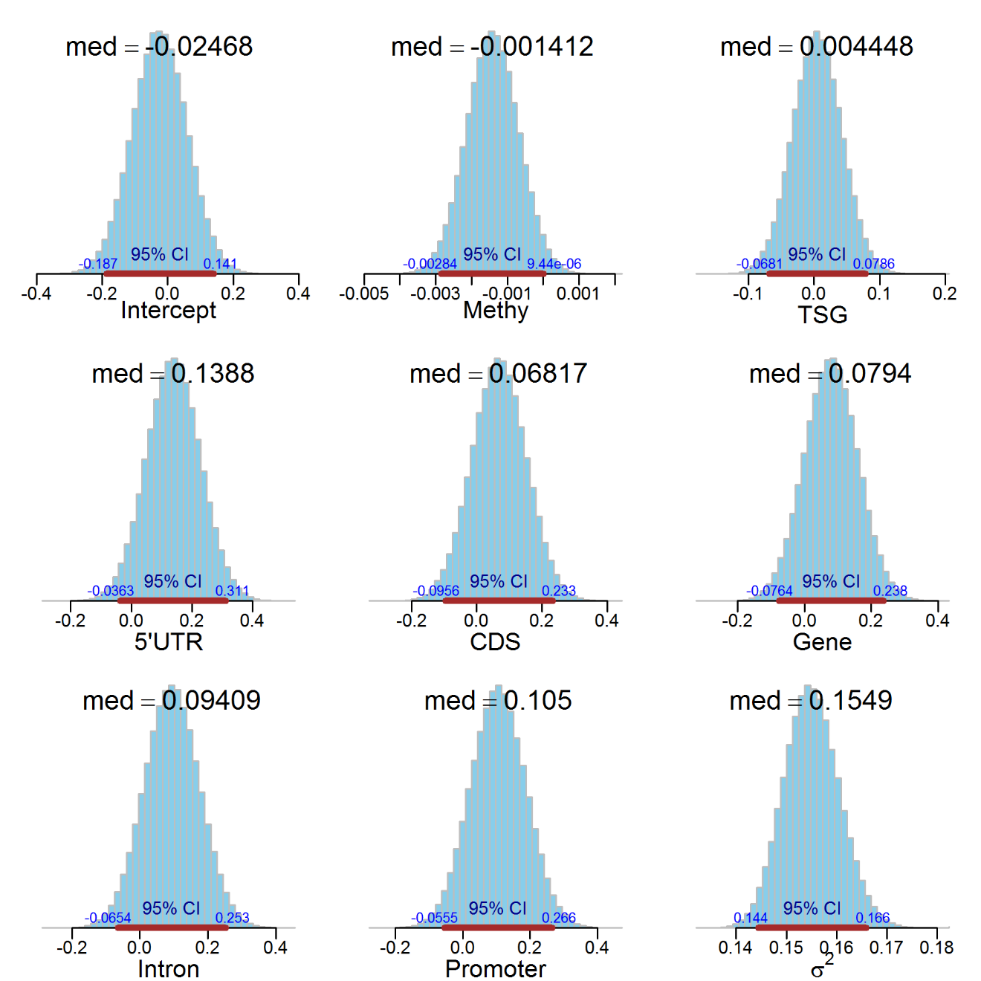


**Figure S7.** Statistics for Gaussian regression for differential gene expression (log2 fold change from RNA-seq) based on methylation level, gene category (tumor suppressor gene or not) and DMR annotation region distribution (gene body, intron, promoter, 5’UTR, 3’UTR and CDS). Each plot lists the median (med) for sampling distribution, together with 95% confidence interval (CI) and start/end positions. The analysis result is for hypo-methylation case.


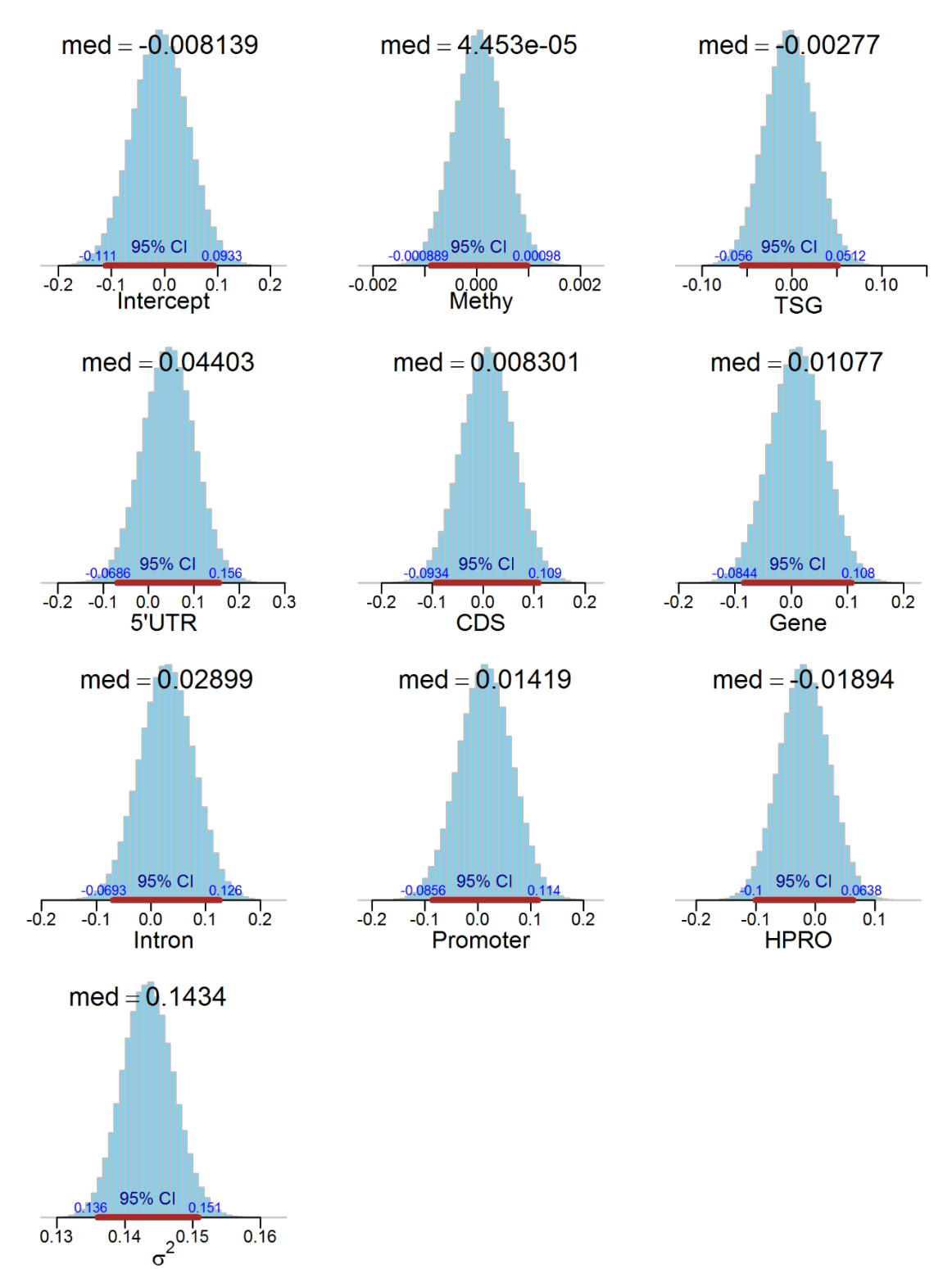


**Figure S8.** Statistics for Gaussian regression for differential gene expression (log2 fold change from RNA-seq) based on methylation level, gene category (tumor suppressor gene or not) and DMR annotation region distribution (gene body, intron, promoter, 5’UTR, 3’UTR and CDS). Each plot lists the median (med) for sampling distribution, together with 95% confidence interval (CI) and start/end positions. The analysis result is for total methylation case (hyper- and hypo-methylation), together with the corresponding variant HPRO introduced into the model.
